# Prevalence of *Mycobacterium tuberculosis* Complex among Wild Rhesus Macaques and 2 Subspecies of Long-Tailed Macaques, Thailand, 2018–2022

**DOI:** 10.3201/eid2903.221486

**Published:** 2023-03

**Authors:** Suthirote Meesawat, Saradee Warit, Yuzuru Hamada, Suchinda Malaivijitnond

**Affiliations:** Author affiliations: Chulalongkorn University Faculty of Science, Bangkok, Thailand (S. Meesawat, S. Malaivijitnond); National Science and Technology Development Agency, Pathumthani, Thailand (S. Warit); Chulalongkorn University National Primate Research Center of Thailand Saraburi, Thailand (Y. Hamada, S. Malaivijitnond)

**Keywords:** Mycobacterium tuberculosis complex, tuberculosis and other mycobacteria, IS6110, bacteria, respiratory infections, macaques, throat swab, zoonoses, Thailand

## Abstract

We identified tuberculosis in 1,836 macaques from 6 wild rhesus (*Macaca mulatta*), 23 common long-tailed (*M. fascicularis fascicularis*), and 6 Burmese long-tailed (*M. fascicularis aurea*) macaque populations in Thailand. We captured, anesthetized, and collected throat, buccal, and rectal swab specimens from the macaques. We screened swabs for *Mycobacterium tuberculosis* complex (MTBC) using insertion sequence 6110–specific nested PCR. We found higher MTBC prevalence at both population and individual levels among *M. mulatta* than *M. fascicularis fascicularis* macaques; all 3 *M. fascicularis aurea* macaque populations were positive for tuberculosis. We found that throat swab specimens provided the best sample medium for detecting MTBC. Our results showed no difference in MTBC prevalence between male and female animals, but a higher percentage of adults were infected than subadults and juveniles. Although we detected no association between frequency of human–macaque interaction and MTBC prevalence, bidirectional zoonotic transmission should be considered a possible public health concern.

Climate change and habitat encroachment by humans have led to overlapping of home ranges of wildlife with human settlements. Subsequently, a vast variety of infectious zoonotic diseases have recently emerged or reemerged, transmitted to humans directly from wildlife or indirectly through domestic animals (*1*). Tuberculosis (TB), caused by *Mycobacterium tuberculosis* bacteria, is an airborne chronic infectious disease with zoonotic potential found among both humans and wildlife (*2*). TB has been with humanity for thousands of years and has not been eliminated by modern medical efforts, despite identification of the causative agent 140 years ago by Dr. Robert Koch. A 2021 World Health Organization report ranked Thailand as a country with one of the highest burdens of human TB (*3*). 

TB has previously been reported among both wild and captive nonhuman primates. Approximately 75% of TB cases in monkeys are caused by *M. tuberculosis*. Among 500 species of nonhuman primates existing throughout the world, rhesus (*Macaca mulatta*) and long-tailed (*M. fascicularis*) macaques have commonly been used as animal models for TB drug and vaccine research because clinical signs and immune responses after MTB infection are similar to those in humans (*4**–**7*). 

Thailand is located at the center of the rhesus and long-tailed macaque distribution ranges, where the 2 species live close together, and comprises part of their interspecific hybridization zone (*8**–**10*). Rhesus macaques are distributed in the north and northeast of Thailand and long-tailed macaques live in central to southern Thailand (*11**–**13*); the hybrid zone, where they cohabit, is within 15°–20°N latitude. It has been proposed that, during earth’s glacial periods, male rhesus macaques introgressed southwards into long-tailed macaque population areas and hybridized with female long-tailed macaques (*9*,*10*). Recently, researchers analyzed the level of genetic admixture between rhesus and long-tailed macaque ancestral populations using autosomal single-nucleotide polymorphism markers. Populations of long-tailed macaques from the northern part of their range carry higher levels of genetic admixture of rhesus ancestry than do southern populations (*10*). 

Thailand has also been the site of core hybridization between common long-tailed (*M. fascicularis fascicularis*) and Burmese long-tailed (*M. fascicularis aurea*) macaques at 8°10′–12°24′N latitude within Thailand (*11*,*14*,*15*). The natural range of Burmese long-tailed macaques spans southward along the Andaman coast to southwestern Thailand (*11*,*15*). During the past decade, researchers have intensively investigated genetic characteristics of Burmese long-tailed macaques using various genetic markers, including partial and whole mitochondrial DNA, Y-chromosome genes TSPY and SRY (*14*,*16*), whole-genome sequences (*17*), and autosomal single-nucleotide polymorphisms (*15*). Those studies indicated that Burmese long-tailed macaques genetically diverge from common long-tailed and rhesus macaques. 

Although both rhesus and long-tailed macaques are widely used for TB research, they differ in their TB pathogenesis, progression, and bacterial burden (*18*). Rhesus macaques are more susceptible than long-tailed macaques to infection after aerosol challenge with *M. tuberculosis* (*19*), and ≈50% of long-tailed macaques showed a clinically latent stage after low-dose exposure to Erdman strain *M. tuberculosis* (*4*). One main concern is that *M. tuberculosis* susceptibility among the 2 macaque species has been tested only in laboratory settings, which do not thoroughly reflect what happens in nature. However, genetic background might also affect levels of susceptibility to *M. tuberculosis *in among different species of macaques. Because of the threat to public health presented by *M. tuberculosis*, we aimed to investigate its prevalence among wild rhesus macaques and 2 subspecies (common and Burmese) of long-tailed macaques whose habitats now overlap with human habitats in Thailand. 

## Methods

### Study Sites and Specimen Collections

During 2018–2022, we captured, sampled, and released 1,836 macaques (189 rhesus, 1,520 common long-tailed, and 127 Burmese long-tailed) from 32 location-defined populations of 2 species of macaques (rhesus and long-tailed), including 2 subspecies of long-tailed macaques (common and Burmese), with distribution ranges in Thailand (*11*,*12*). We identified species and subspecies on the basis of morphologic characteristics, including pelage color, relative tail length, check hair pattern, and head crest (*11*,*12*,*14*,*20*). We recorded the habitat types (temples and tourist attraction sites) where we found them and the frequency of interaction (daily, weekly, monthly, or rarely) with humans (Table 1). 

**Table 1:** Macaque species, locations, geographic coordinates, habitat types, and frequency of interaction with humans from study of macaques infected with *Mycobacterium tuberculosis* complex, Thailand, 2018–2022

Species‎ (common name)	Location	Latitude N, longitude E	Habitat type*	Interaction frequency†
*Macaca mulatta* (rhesus)	Wat Phra Phutthabat Pha Ruea	20°10′, 100°03′	A	3
	Wat Tham Mueang On	18°47′, 99°14′	B	3
	Wat Tham Thorani Siri Ram	17°11′, 99°33′	C	0
	Ban Sang School	17°51′, 103°57′	B	2
	Ban Phon Kor	17°64′, 104°34′	A	3
	Wat Tham Erawan	17°20′, 101°59′	A	3
*M. fascicularis fascicularis* (common long-tailed)	Wat Haad Moon	16°30′, 100°16′	C	3
	Wat Ta Sung Tai	15°94′, 99°95′	A	3
	Kao Nor	15°57′, 99°52′	B	2
	Wat Tham Thep Ban Dan	15°44′, 101°02′	C	2
	Wat Mueang Khaen Yai	15°36′, 104°21′	C	3
	Muang Ling Ban Wan	15°38′, 104°18′	B	2
	Wat Ku Phra Ko Na	15°33′, 103°49′	A	3
	Wat Phikun Ngam	15°27′, 100°05′	C	2
	Suan Ling Garden	14°98′, 100°23′	A	3
	Lopburi	14°80′, 100°61′	B	3
	Phar Phothisat	14°57′, 101°14′	A	3
	Wat Kai	14°50′, 100°52′	A	3
	Phra Phutthabat Noi	14°39′, 100°58′	C	2
	Khao Laem Pu Chao	13°39′, 100°52′	B	3
	Wat Tham Khao Chakan	13°39′, 102°05′	A	3
	Wat Tham Khao Cha Ang	13°12′, 101°39′	A	3
	Wat Khao Cha Ang	13°11′, 101°31′	A	3
	Wat Khao Wong Khot	12°52′, 101°49′	A	3
	Kao Ngu	13°34′, 99°46′	B	2
	Wat Kao Tharmon	13°02′, 99°57′	A	3
	Wat Suwan Kuha	8°25′, 98°28′	A	3
	Wat Khao Keaw Wichian	8°12′, 100°05′	C	1
	Khao Chaison	7°27′, 100°07′	B	3
*M. fascicularis aurea* (Burmese long-tailed)	Tham Pra Khayang	10°19′, 98°45′	B	1
	World War Museum	10°10′, 98°43′	B	0
	Mangrove Forest Research Center	9°87′, 98°60′	B	1

*A, temple (tourist site); B, nontemple tourist site; C, temple (nontourist site). †0, rarely; 1, monthly; 2, weekly; 3, daily.

We captured the macaques in iron mesh traps and anesthetized them using an intramuscular injection of 2–5 mg/kg body weight of tiletamine/zolazepam (Virbac, https://us.virbac.com) mixed with 20–50 μg/kg body weight of (dex)medetomidine hydrochloride (Zoetis, https://www.zoetis.com) (*13*,*15*). We recorded sex, body weight, and rectal temperature and estimated age on the basis of dental eruption patterns (*21*); we also attached numeric identification tags to the animals’ legs. Using cotton swabs, we took throat swab specimens from between the base of the tongue and the soft palate, buccal swab specimens from the bulge of the cheek pouches, and rectal swab specimens from the anus. We stored swabs at room temperature in 1.5 mL of sterile lysis buffer (0.5% wt/vol sodium dodecyl sulfate, 100 mmol pH 8.0 ethylenediaminetetraacetic acid, 100 mmol pH 8.0 tris aminomethane hydrochloride, and 10 mmol NaCl) (*9*,*10*) until time of genomic DNA extraction. We collected throat and buccal swab specimens from 1,836 macaques in all 32 populations but collected rectal swab specimens from only 1,681 in 28 populations (Table 1). After we had collected all biologic specimens, we administered an intramuscular injection of atipamezole hydrochloride (Zoetis) at the same volume as the (dex)medetomidine hydrochloride anesthetic dose; after their recovery from anesthesia, we released the macaques back to their habitats.

The National Primate Research Center of Thailand-Chulalongkorn University (NPRCT-CU) Animal Care and Use Committees approved all animal procedures (protocol review no. 2075007). The Thailand Department of the National Parks, Wildlife and Plant Conservation approved the protocols for capturing and collecting specimens from macaques. 

### DNA Extraction and MTB Detection Using Nested PCR

We incubated swab samples at 70°C for 1 h with 50 μL of 30 mg/mL lysozyme solution (SERVA, https://www.serva.de) and extracted genomic DNA using an automated QIAsymphony Virus/Pathogen Mini Kit (QIAGEN, https://www.qiagen.com). We measured the concentration of extracted DNA using QIAGEN QIAxpert. We amplified extracted DNA using nested PCR with MTBC insertion sequence (IS) 6110–specific primers, according to a protocol developed for specimens from humans (*22*). We amplified the MTBC IS6110 DNA (*23*) for 2 rounds. For the first round, we used Tb 294 (5′-GGACAACGCCGAATTGCGAAGGGC-3′) and Tb 850 (5′-TAGGCGTCGGTGACAAAGGCCACG-3′) primers to achieve a 580-bp amplicon. For the second-round nested PCR, we used Tb 505 (5′-ACGACCACATCAACC-3′) and Tb 670 (5′-AGTTTGGTCATCAGCC-3′) primers to achieve a 181-bp amplicon. The 25-μL PCR reaction mixture consisted of 17.4 μL deionized distilled water, 0.5 μL of each primer (0.4 pmol/μL), 2 μL DNA template, 4.6 μL TaKaRa Ex Taq Hot Start Version Kit, 2.5 μL 10X Ex Taq Buffer (Mg^2+^ plus), and 2.0 μL each of 2.5 mmol deoxynucleoside triphosphate and 0.125 μL TaKaRa Ex Taq Hot (TaKaRa Bio, https://www.takarabio.com).

We ran the PCR using Applied Biosystems Verti 96-Well Thermal Cyclers (Thermo Fisher Scientific, https://www.thermofisher.com). For the first round of PCR, we set thermal cycling at 98°C for 1 min, followed by 30 cycles at 93°C for 20 s, 65°C for 30 s, 72°C for 1 min, and 72°C for 10 min. We amplified 1 μL of the first-round PCR product in the second round at 98°C for 1 min, followed by 30 cycles at 93°C for 20 s, 48°C for 30 s, 72°C for 30 s, and 72°C for 10 min. We visualized the 181-bp PCR products using 2% wt/vol agarose gel electrophoresis and stained them with Invitrogen SYBR Safe DNA gel stain (Thermo Fisher). 

### Specificity and Sensitivity of MTBC Detection Using IS6110 Nested PCR

**Figure 1:**
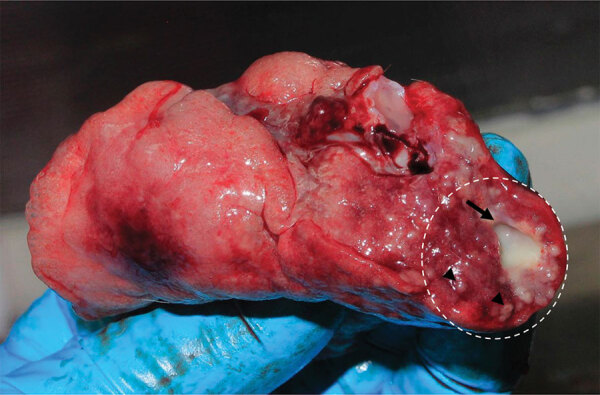
Biopsied lung of a naturally *Mycobacterium tuberculosis*–infected common long*-*tailed macaque collected in Thailand. Within the white dashed circle, arrow indicates granuloma and arrowheads indicate multiple*-*sized granulomas diffused within the lung parenchyma.

We validated specificity of the MTBC nested PCR with IS6110-specific primers using granuloma lung tissues from a naturally *M. tuberculosis*–infected long-tailed macaque (Figure 1). We confirmed *M. tuberculosis* infection in the macaque by mycobacterium culture and interferon gamma release assay, using methods reported elsewhere (*24*). We collected and extracted granuloma lung tissues for genomic DNA using a QIAGEN Virus/Pathogen Mini Kit. We purified the products obtained from 181-bp nested PCR testing of the naturally *M. tuberculosis*–infected macaque and the 19 nested PCR–positive samples randomly selected from populations of wild rhesus macaques from Ban Phon Kor and Ban Sang School and Burmese long-tailed macaques from Tham Pra Khayang and Mangrove Forest Research Center using the GenUP Exo Sap Kit (Biotechrabbit, https://www.biotechrabbit.com) and submitted the samples to Macrogen (https://www.macrogen.com) for DNA sequencing. We aligned nucleotide sequences with published *M. tuberculosis* sequences accessed from GenBank using MEGA X software (*25*). After all amplicons from the *M. tuberculosis* sequences from macaques in our study showed 100% homology with published sequences, we used an IS6110*-*specific nested PCR protocol to determine the sensitivity of the *M. tuberculosis* nested PCR technique, using genomic DNA of the *M. tuberculosis* H37Rv (ATCC27294) strain, serially diluted (1:10) from 1 ng to 1 fg (*26*). We designated the lowest concentration of *M. tuberculosis* H37Rv that we could detect by nested PCR as the limit of detection (LOD) for this technique. 

### Data Analyses

We calculated prevalence rate and 95% CIs for differences in MTBC shedding stratified by species and subspecies, sex, and age of macaques. Using Pearson χ^2^ tests, we analyzed associations between MTBC prevalence and macaque species and subspecies, sex and age, and between MTBC prevalence and frequency of human–macaque interaction. Using Pearson correlation analysis, we analyzed correlations between MTBC prevalence and bodily site (throat, buccal and rectal) of specimen collection and used SPSS Statistics 28 for Mac (IBM, https://www.ibm.com) to analyze data. We used p≤0.05 to indicate statistical significance. 

## Results

### Specificity and Sensitivity of MTBC Detection Using IS6110 Nested PCR

The 181-bp nucleotide sequences from granuloma lung tissues and 19 nested PCR–positive samples from a naturally *M. tuberculosis*–infected macaque all showed 100% similarity with the MTB H37Rv complete genome (GenBank accession no. NC_000962.3) (data not shown). A BLASTn (https://blast.ncbi.nlm.nih.gov/Blast.cgi) search using the NCBI-registered complete genomes indicated that the specific 181-bp nucleotide sequence obtained from the IS6110-specific nested PCR (574 *M. tuberculosis*; 8 *M. bovis* BCG; 3 *M. canettii*; 2 each *M. bovis* and *M. africanum*; and 1 each *M. caprae*, *M. microti*, and *M. orygis* strains) could be detected in the MTBC. Thus, we interpreted the results from the IS6110-specific nested PCR in this study as positive for MTBC. 

**Figure 2:**
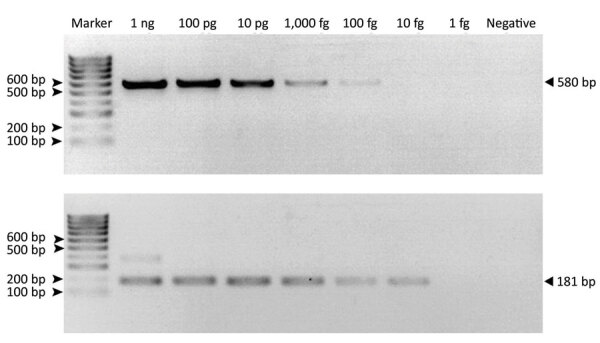
Limit of detection of *Mycobacterium tuberculosis* complex in the first (A) (580-bp) and second (B) (181-bp) rounds of IS6110-specific nested PCR of samples from free*-*ranging macaques, Thailand, 2018–2022. We could not visualize 580-bp PCR products from resolved 2% wt/vol agarose gel electrophoresis in the first round but detected a positive result from the second-round nested PCR with a 181-bp amplicon.

The optimized MTBC nested PCR conditions of the H37Rv DNA (ATCC27294) strain showed LOD values of 100 fg/μL in the first round (580-bp product) and 10 fg/μL in the second round (181-bp product) (Figure 2). Because MTBC concentration was low in some macaques, we could not visualize 580-bp PCR products from resolved 2% wt/vol agarose gel electrophoresis but detected a positive result from the second-round nested PCR with a 181-bp amplicon. In the second round in this study, we found 71/192 MTBC-positive specimens overall: 21/80 for rhesus, 36/93 for common long-tailed, and 14/19 for Burmese long-tailed macaques. 

### Prevalence of MTBC in Rhesus Macaques and 2 Subspecies of Long***-***Tailed Macaques

**Figure 3:**
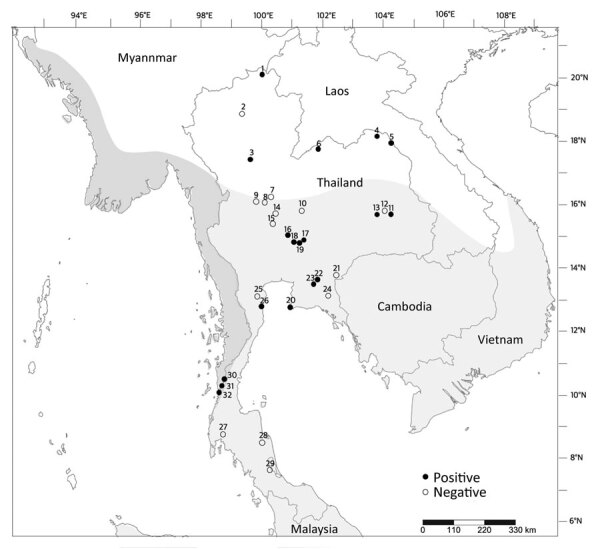
Locations, species, and *Mycobacterium tuberculosis* complex status in free*-*ranging macaques, Thailand, 2018–2022. White area on map shows the range of rhesus macaques; light gray, common long-tailed macaques; and dark gray, Burmese long-tailed macaque. Numbers indicate locations where we found macaques: 1, Wat Phra Phutthabat Pha Ruea; 2, Wat Tham Mueang On; 3, Wat Tham Thorani Siri Ram; 4, Ban Sang School; 5, Ban Phon Kor; 6, Wat Tham Erawan; 7, Wat Haad Moon; 8, Wat Ta Sung Tai; 9, Kao Nor; 10, Wat Tham Thep Ban Dan; 11, Wat Mueang Khaen Yai; 12, Muang Ling Ban Wan; 13, Wat Ku Phra Ko Na; 14, Wat Phikun Ngam; 15, Suan Ling Garden; 16, Lopburi; 17, Phar Phothisat; 18, Wat Kai; 19, Phra Phutthabat Noi; 20, Khao Laem Pu Chao; 21, Wat Tham Khao Chakan; 22, Wat Tham Khao Cha Ang; 23, Wat Khao Cha Ang; 24, Wat Khao Wong Khot; 25, Kao Ngu; 26, Wat Kao Tharmon; 27, Wat Suwan Kuha; 28, Wat Khao Keaw Wichian; 29, Khao Chaison; 30, Tham Pra Khayang; ; 31, World War Museum; 32, Mangrove Forest Research Center.

We detected MTBC IS6110 DNA among all 3 (100%) Burmese long-tailed macaque populations, higher than for either rhesus (5/6, 83.3%) or common long-tailed (10/23, 43.5%) macaque populations (Table 2; Figure 3). We found MTBC IS6110 DNA in 128/1,836 (7.0%) throat, 41/1,836 (2.3%) buccal, and 23/1,681 (1.4%) rectal swab samples (Table 2). When we analyzed specimens from all body collection sites, correlation was significant between 1,836 throat and buccal (r^2^ *=* 0.04; p *=* 0.01), 1,681 throat and rectal (r^2^ = 0.01; p *=* 0.01), and 1,681 buccal and rectal swab samples (r^2^ *=* 0.07; p *=* 0.01). However, when we analyzed only MTBC-positive results, we found significant correlations only between 152 throat and buccal (r^2^ *=* 0.29; p *=* 0.01), and 99 throat and rectal swab samples (r^2^ *=* 0.30; p *=* 0.01), but not between 45 buccal and rectal swab samples (r^2^ *=* 0.004; p *=* 0.54).

**Table 2:** Detection of MTBC in throat, buccal, and rectal swab specimens tested by using IS6110-specific nested PCR in 32 populations of macaques, by swab site and location found, Thailand, 2018–2022*

Species (common name)	Location	Throat and buccal/rectal specimens	MTBC-PCR detected in swabs, no. (%)							MTBC-positive (%)
			Throat	Buccal	Rectal	Throat + buccal	Throat + rectal	Buccal + rectal	All 3	
*Macaca mulatta* (rhesus)	Wat Phra Phutthabat Pha Ruea	57/57	2 (3.5)	1 (1.8)	2 (3.5)	0	1 (1.8)	0	0	4 (7.0)
	Wat Tham Mueang On	10/10	0	0	0	0	0	0	0	0
	Wat Tham Thorani Siri Ram	1/1	0	0	1 (100)	0	0	0	0	1 (100.0)
	Ban Sang School	42/0	29 (69.0)	4 (9.5)	NA	4 (9.5)	0	0	0	29 (69.0)
	Ban Phon Kor	19/0	12 (63.2)	3 (15.8)	NA	2 (10.5)	0	0	0	13 (68.4)
	Wat Tham Erawan	60/60	23 (38.3)	3 (5.0)	0	3 (5.0)	0	0	0	23 (38.3)
	Total	189/128	66 (34.9)	11 (5.8)	3 (2.3)	9 (4.8)	1 (0.8)	0	0	70 (37.0)
*M. fascicularis fascicularis* (common long-tailed)	Wat Haad Moon	34/34	0	0	0	0	0	0	0	0
	Wat Ta Sung Tai	60/60	0	0	0	0	0	0	0	0
	Kao Nor	77/77	0	0	0	0	0	0	0	0
	Wat Tham Thep Ban Dan	55/55	0	0	0	0	0	0	0	0
	Wat Mueang Khaen Yai	60/60	5 (8.3)	12 (20.0)	8 (13.3)	1 (1.7)	0	4 (6.7)	1 (1.7)	20 (33.3)
	Muang Ling Ban Wan	4/4	0	0	0	0	0	0	0	0
	Wat Ku Phra Ko Na	77/77	9 (11.7)	0	2 (2.6)	0	2 (2.6)	0	0	7 (9.1)
	Wat Phikun Ngam	11/11	0	0	0	0	0	0	0	0
	Suan Ling Garden	148/148	0	0	0	0	0	0	0	0
	Lopburi	91/91	2 (2.2)	1 (1.1)	0	1 (1.1)	0	0	0	1 (1.1)
	Phar Phothisat	304/304	7 (2.3)	1 (0.3)	0	0	0	0	0	8 (2.6)
	Wat Kai	60/60	5 (8.3)	0	0	0	0	0	0	5 (8.3)
	Phra Phutthabat Noi	118/118	5 (4.2)	0	0	0	0	0	0	5 (4.2)
	Khao Laem Pu Chao	10/10	1 (10.0)	0	1 (10.0)	0	0	0	0	2 (20.0)
	Wat Tham Khao Chakan	50/50	0	0	0	0	0	0	0	0
	Wat Tham Khao Cha Ang	36/36	9 (25.0)	5 (13.9)	1 (2.8)	2 (5.6)	0	1 (2.8)	0	13 (36.1)
	Wat Khao Cha Ang	15/15	6 (40.0)	4 (26.7)	1 (6.7)	1 (6.7)	0	1 (6.7)	0	9 (60.0)
	Wat Khao Wong Khot	47/47	0	0	0	0	0	0	0	0
	Kao Ngu	71/71	0	0	0	0	0	0	0	0
	Wat Kao Thamon	72/72	7 (9.7)	1 (1.4)	0	0	0	0	0	8 (11.1)
	Wat Suwan Kuha	28/28	0	0	0	0	0	0	0	0
	Wat Khao Keaw Wichian	33/33	0	0	0	0	0	0	0	0
	Khao Chaison	59/59	0	0	NA	0	0	0	0	0
	Total	1,520/1,461	56 (3.7)	24 (1.6)	13 (0.9)	5 (0.3)	2 (0.1)	6 (0.4)	1 (0.1)	78 (5.1)
*M. fascicularis aurea* (Burmese long-tailed)	Tham Pra Khayang	40/40	2 (5.0)	1 (2.5)	3 (7.5)	0	1 (2.5)	0	0	5 (12.5)
	World War Museum	52/52	0	1 (1.9)	4 (7.7)	0	0	0	0	5 (9.6)
	Mangrove Forest Research Center	35/0	4 (11.4)	4 (11.4)	NA	0	0	0	0	8 (22.9)
	Total	127/92	6 (4.7)	6 (4.7)	7 (7.6)	0	1 (1.1)	0	0	18 (14.2)
All	Total	1,836/1,681	128 (7.0)	41 (2.3)	23 (1.3)	14 (0.8)	4 (0.2)	6 (0.4)	1 (0.1)	166 (9.0)

*MTBC, *Mycobacterium tuberculosis* complex; NA, not available.

Because we detected MTBC-positive cases most frequently from throat swab specimens, we further analyzed the 16 location-specific populations that showed MTBC-positive results from throat swab specimens: Wat Phra Phutthabat Pha Ruea, Ban Sang School, Ban Phon Kor, and Wat Tham Erawan for rhesus macaques; Wat Mueang Khaen Yai, Wat Ku Phra Ko Na, Lopburi, Phar Phothisat, Wat Kai, Phra Phutthabat Noi, Khao Laem Pu Chao, Wat Tham Khao Cha Ang, Wat Khao Cha Ang, and Wat Kao Tharmon for common long-tailed macaques; and Tham Pra Khayang and Mangrove Forest Research Center for Burmese long-tailed macaques (Tables 2, 3). Based on these data, MTBC prevalence was significantly higher (χ^2^ = 253.95; p = 0.01) among rhesus (37.1% [95% CI 30.0%–44.2%]; 66/178 in 4 populations) than Burmese long-tailed (8.0% [95% CI 1.9%–14.1%]; 6/75 in 2 populations) and common long-tailed (6.6% [95% CI 5.0%–8.3%]; 56/843 in 10 populations) macaques. Most MTBC-positive common long-tailed macaques inhabited the central region of Thailand at 13°02′–15°36′N latitude (Figure 3).

**Table 3:** Detection of MTBC in throat swabs using IS6110-specific nested PCR in 32 populations of macaques, by sex and location found, Thailand, 2018–2022*

Species (common name)‎	Location	No. positive/total no. (%)	MTBC PCR detected in throat swab specimens, no. positive/total no. (%)						
			M age groups†				F age groups†		
			Adult	Subadult	Juvenile		Adult	Subadult	Juvenile
*Macaca mulatta* (rhesus)	Wat Phra Phutthabat Pha Ruea	2/57 (3.5)	0/2	0/10	1/18 (5.6)		0/4	0/10	1/13 (7.7)
	Ban Sang School	29/42 (69.0)	6/9 (66.7)	4/5 (80.0)	3/6 (50.0)		11/15 (73.3)	3/5 (60.0)	2/2 (100.0)
	Ban Phon Kor	12/19 (63.2)	4/5 (80.0)	2/3 (66.7)	3/5 (60.0)		1/3 (33.3)	1/1 (100.0)	1/2 (50.0)
	Wat Tham Erawan	23/60 (38.3)	2/3 (66.7)	3/10 (30.0)	2/10 (20.0)		7/11 (63.6)	6/18 (33.3)	3/8 (37.5)
	Total	66/178 (37.1)	12/19 (63.2)	9/28 (32.1)	9/39 23.1)		19/33 (57.6)	10/34 (29.4)	7/25 (28.0)
*M. fascicularis fascicularis* (common long-tailed)	Wat Mueang Khaen Yai	5/60 (8.3)	0/2	0/15	1/8 (12.5)		3/22 (13.6)	1/11 (9.1)	0/2
	Wat Ku Phra Ko Na	9/77 (11.7)	1/13 (7.7)	3/22 (13.6)	0/11		3/13 (23.1)	1/9 (11.1)	1/9 (11.1)
	Lopburi	2/91 (2.2)	0/21	0/17	0/9		1/29 (3.4)	1/11 (9.1)	0/4
	Phar Phothisat	7/304 (2.3)	3/51 (5.9)	2/35 (5.7)	0/27		1/102 (1.0)	1/57 (1.8)	0/32
	Wat Kai	5/60 (8.3)	3/25 (12.0)	0/15	0/0		2/10 (20.0)	0/9	0/1
	Phra Phutthabat Noi	5/118 (4.2)	2/2 (100.0)	1/5 (20.0)	1/52 (1.9)		0/5	1/12 (8.3)	0/42
	Khao Laem Pu Chao	1/10 (10.0)	1/3 (33.3)	0/3	0/0		0/0	0/2	0/2
	Wat Tham Khao Cha Ang	9/36 (25.0)	1/9 (11.1)	1/2 (50.0)	3/10 (30.0)		0/3	2/6 (33.3)	2/6 (33.3)
	Wat Khao Cha Ang	6/15 (40.0)	4/10 (40.0)	0/0	0/0		0/0	1/2 (50.0)	1/3 (33.3)
	Wat Kao Tharmon	7/72 (9.7)	0/23	2/20 (10.0)	1/6 (16.7)		3/11 (27.3)	1/9 (11.1)	0/3
	Total	56/843 (6.6)	15/159 (9.4)	9/134 (6.7)	6/123 (4.9)		13/195 (6.7)	9/128 (7.0)	4/104 (3.9)
*M. fascicularis aurea* (Burmese long-tailed)	Tham Pra Khayang	2/40 (5.0)	1/15 (6.7)	0/4	1/5 (20.0)		0/6	0/7	0/3
	Mangrove Forest Research Center	4/35 (11.4)	2/10 (20.0)	1/7 (14.3)	1/9 (11.1)		0/2	0/4	0/3
	Total	6/75 (8.0)	3/25 (12.0)	1/11 (9.1)	2/14 (14.3)		0/8	0/11	0/6
All	Total	128/1,096 (11.7)	30/203 (14.8)	19/173 (11.0)	17/176 (9.7)		32/236 (13.6)	19/173 (11.0)	11/135 (8.1)

*MTBC, *Mycobacterium tuberculosis* complex. †Adult, >6 y; subadult, 3–6 y; juvenile, <3 y.

We futher analyzed the data for any correlation between MTBC prevalence and age and sex. We stratified data into 3 groups on the basis of age estimated from dental eruption at the time of capture; we classified both male and female macaques >6 years as adults, 3–6 years as subadults, and <3 years as juveniles (Tables 3, 4). MTBC shedding status detected from throat swab specimens was significantly higher among adult positive case-patients than other age groups of the same species: 47.0% (31/66) for adult rhesus macaques, 50.0% (28/56) for adult common long-tailed macaques, and 50.0% (3/6) for adult Burmese long-tailed macaques (χ^2^ = 0.14; p = 0.01). When we considered data on MTBC prevalence by sex of positive case-patients, the difference between males (51.6%, 66/128) and females (48.4%, 62/128) was not significant (χ^2^ = 0.94; p = 0.33). We also found no significant difference by swab collection site between percentages of MTBC-positive macaques and frequency of human interaction (χ^2^ = 6.76, p = 0.08 for throat; χ^2^ = 5.41, p = 0.14 for buccal; and χ^2^ = 3.27, p value = 0.11 for rectal swab samples). 

**Table 4:** Detection of *Mycobacterium tuberculosis* complex in throat swabs using IS6110-specific nested PCR in 32 populations of macaques, Thailand, 2018–2022*

Species (common name)	Age group	Male	Female	Total
		Positive, % (95% CI)	Positive, % (95% CI)	Positive, % (95% CI)
*Macaca mulatta* (rhesus)	Adult	12, 18.2 (8.9–27.5)	19, 28.8 (17.9–39.7)	31, 47.0 (24.4–47.6)
	Subadult	9, 13.6 (5.4–21.9)	10, 15.2 (6.5–23.8)	19, 28.8 (17.9–39.7)
	Juvenile	9, 13.6 (5.4–21.9)	7, 10.6 (0.8–13.2)	16, 24.2 (13.9–34.6)
	Total	30, 45.5 (33.4–57.5)	36, 54.5 (24.4–47.6)	**66, 100.0 (100.0–100.0)**
*M. fascicularis fascicularis* (common long-tailed)	Adult	15, 26.8 (15.2–38.4)	13, 23.2 (12.1–34.3)	28, 50.0 (36.9–63.1)
	Subadult	9, 16.1 (6.5–25.7)	9, 16.1 (6.5–25.7)	18, 32.1 (19.9–44.4)
	Juvenile	6, 10.7 (2.6–18.8)	4, 7.1 (0.4–13.9)	10, 17.9 (7.8–27.9)
	Total	30, 53.6 (40.5–66.6)	26, 46.4 (33.4–59.5)	56, 100.0 (100.0–100.0)
*M. fascicularis aurea* (Burmese long-tailed)	Adult	3, 50.0 (10.0–90.0)	0	3, 50.0 (10.0–90.0)
	Subadult	1, 16.7 (0.0–46.5)	0	1, 16.7 (0.0–46.5)
	Juvenile	2, 33.3 (0.0–71.1)	0	2, 33.3 (0.0–71.1)
	Total	6, 100.0 (100.0–100.0)	0	6, 100.0 (100.0–100.0)
All	Total	66, 51.6 (42.9–60.2)	62, 48.4 (39.8–57.1)	128, 100.0 (100.0–100.0)
*Bold indicates significant difference (p <0.05) from other species				

## Discussion

Intradermal tuberculin skin test (TST) is used globally to detect TB in macaques. Testers intradermally inject tuberculin antigens at the edge of the animal’s upper eyelid and look for an immune response (erythema or edema) at 24, 48, and 72 hours after injection (*27*). Disadvantages of TST include that it depends on an interpreter’s subjective judgment and is time-consuming and impractical for field study in free-ranging or wild macaques. Therefore, we collected swabs of biologic specimens from common sites of MTBC shedding—throat, cheeks, and rectum—for screening. To test samples, we modified a nested PCR technique previously developed for clinical specimens using IS6110-specific primers (*22*) and used it to detect MTBC among free-ranging rhesus and long-tailed macaques. Results in samples taken from multiple sites (*22*,*28*,*29*) revealed a higher prevalence of MTBC DNA in throat than in buccal and rectal swab specimens. Thus, we determined that, because of their higher efficiency, throat swab specimens should be used for collecting specimens to detect MTBC shedding in free*-*ranging macaques. Because of low MTBC concentration in the collected specimens, most 580-bp band PCR amplifications in the first round were undetected; however, we could clearly identify the 181-bp PCR amplicons in agarose gel in the second round. LOD for the second-round nested PCR, 10 fg/μL, was 10-fold lower than in the first round, 100 fg/μL, similar to findings reported elsewhere (*26*). Although this nested PCR had the advantage of higher sensitivity, the possibility of false-positive results from contaminated DNA or DNA carryover, mainly amplicons, should be considered. To avoid cross-contamination or carryover, key processes, such as template preparation, specimen handling, and preparation of PCR mixture, should be performed in different PCR biosafety cabinets and should include negative controls. 

In a previous report, laboratory-tested rhesus macaques were more susceptible to MTBC infection than long-tailed macaques (*19*); likewise, in our study, free-ranging rhesus macaques in Thailand showed higher MTBC prevalence than did common long-tailed macaques, both in the number of populations and individual macaques infected. The laboratory-tested rhesus macaques exhibited illness, had respiratory tract granuloma develop, and returned to activity after a short duration. In contrast, free-ranging rhesus macaques in our study showed no signs of illness (*5*,*6*,*30*). One possible explanation for this difference is that the rhesus macaques in Thailand had a genetic admixture of long-tailed macaque ancestry, which made them more resistant to MTBC. For example, rhesus macaques in the population at Ban Sang School, which carried up to an 18% genetic admixture of long-tailed macaque ancestry, had a high percentage, 29%, of MTBC prevalence (*10*). Considering the high MTBC prevalence in rhesus macaques and frequent interaction with humans among some populations, there is a need for practices such as avoiding direct contact with macaques and conveying information to local residents about the spread of TB from macaques. Local residents should also be monitored for bidirectional transmission of MTBC. 

In light of usual rates of MTBC infection among long-tailed macaques, it is notable that all 3 populations of Burmese long-tailed macaques were MTBC positive, in spite of less frequent contact with humans, rare or monthly, than for populations of common long-tailed macaques. In particular, the World War Museum and Mangrove Forest Research Center populations in Ranong Province in southern Thailand reside in mangrove forests and roam freely for invertebrate foods. Unique genetic characteristics among Burmese long-tailed macaques, identified through intensive genetic studies (*14*–*17*), might factor into their high MTBC prevalence. More research should be conducted on TB infection among Burmese long-tailed macaques, especially about mechanisms of infection and disease progression related to genetic factors, because the species might prove to be an alternative animal model for TB research. Of note, we did not see higher MTBC positivity among northern common long-tailed macaques, even though they carry a higher genetic admixture of rhesus ancestry than their southern relatives (*10*). Thus, factors other than hybridization should be considered when assessing MTBC prevalence among common long-tailed macaques. 

MTBC shedding in 10/23 common long-tailed macaques populations in our study differed from a report published elsewhere (*31*), in which *Mycobacterium* spp. were not detected in any of 649 free-living common long-tailed macaques from 26 locations (*31*). This discrepancy might be because of differences between studies in sampling locations, specimen collection methods, DNA extraction processes, and PCR performance. Although the researchers in that study used IS6110-specific primer sets, they did not conduct a nested PCR, so the sensitivity of their method might have been insufficient to detect low-level shedding of MTBC in macaques. 

Although nested PCR is highly sensitive and less time-consuming than other methods, it can detect TB only during the active shedding stage of infection (*32*,*33*). Other diagnostic tools, such as TST or TB blood test, are still required to detect TB during latent or nonshedding active stages. Another disadvantage of the nested PCR approach is the invasive methods needed to catch and anesthetize macaques to collect specimens, which in some locations, such as mangrove forests (e.g., Mangrove Forest Research Center) or high cliffs (e.g., Wat Suwan Kuha), are also impractical. A noninvasive technique to collect samples should be developed, such as using a rope bait method to collect pathogens inside the oral cavity (*33*) or analyzing samples from recently dropped feces (*34*). On the basis of our results showing comparable prevalence of MTBC infection between male and female macaques and higher prevalence among adults than other age groups, specimen collection efforts could focus on adults in free-ranging macaque populations if only preliminary results are needed. 

Because MTBC-infected wild macaques could be reservoirs of the pathogen and it might be transmitted back to humans, bidirectional zoonotic transmission should be considered, especially for populations in which the MTBC-infected macaques interact with humans on a daily basis. In a worst-case scenario, MTBC bacteria could mutate after infecting a macaque, then be transmitted back to humans; existing commercial antimycobacterial agents might be ineffective for treating the mutated pathogen (*35*). Experience with SARS-CoV-2 virus (*36*) and other emerging and reemerging pathogens transmitted from wildlife to humans has provided lessons about surveilance for and control of emerging public health threats that can be applied to managing potential threats to public health from MTBC. 
